# Congenital Hypertrophic Pyloric Stenosis in a Preterm Dizygotic Female Twin Infant: Case Report

**DOI:** 10.3390/children9040573

**Published:** 2022-04-17

**Authors:** Besiana P. Beqo, Alireza Basharkhah, Emir Q. Haxhija

**Affiliations:** Department of Pediatric and Adolescent Surgery, Medical University of Graz, Auenbruggerplatz 34, A-8036 Graz, Austria; besiana.beqo@medunigraz.at (B.P.B.); alireza.basharkhah@medunigraz.at (A.B.)

**Keywords:** hypertrophic pyloric stenosis, congenital, genetic, twins, preterm infants, diagnostics

## Abstract

Infants with hypertrophic pyloric stenosis are usually diagnosed at about 3 to 8 weeks of age. The clinical onset of symptoms in preterm babies is observed normally at a later age than in term or post-term newborns. This report describes a rare case of a 2-day old preterm twin girl presenting with drinking laziness and recurrent vomiting. Five days after the beginning of symptoms and after several studies, including an upper gastrointestinal contrast study, the diagnosis of hypertrophic pyloric stenosis was made and confirmed at surgery. The postoperative course was uneventful. Interestingly, the mother of the child herself had a history of postnatal surgery on her fifth day of life due to congenital hypertrophic pyloric stenosis. To our best knowledge, this is the first report in the literature describing congenital hypertrophic pyloric stenosis in a mother and her child.

## 1. Introduction

Hypertrophic pyloric stenosis (HPS) is the most frequent cause of bile-free projectile vomiting in newborns. It is characterized by the functional obstruction of the stomach due to hypertrophy of the pyloric muscle of still unknown etiology. Symptoms usually occur between the third and eighth week of life and lead to weight loss, dehydration, and alkalosis. Caucasian babies have the highest risk of being affected, with a male-to-female ratio of 4:1 [1]. HPS shows a robust familial aggregation and a correlation with other diseases such as anomalies of the heart, urological tract, hiatal hernia, and a number of syndromes [2,3]. 

An early onset of HPS presenting soon after birth has only scarcely been reported. In a large case–control study, Demian et al. showed that only 6% of infants diagnosed with HPS were less than 14 days old [4]. These infants had a significantly higher positive family history for HPS when compared to infants who presented with HPS after day 14 of life. Besides the early presentation of HPS, also the late presentation of HPS has been reported in the literature as a rare event [5]. Although genetic factors are commonly discussed in the etiopathogenesis of HPS, this matter still remains unresolved and environmental factors are presently considered equally important [6,7,8,9].

We present a case of an early postnatal HPS with the distinctive feature that the mother of the child herself also had an early postnatal surgery on her fifth day of life due to HPS. 

## 2. Case Report

A 32-year-old internally healthy, non-smoking woman delivered vaginally in the 36th week of a non-complicated dizygotic twin pregnancy first a healthy boy weighing 2940 g and 7 min later a female child weighing 2400 g. Whereas the baby boy remained healthy, the baby girl showed increased drinking laziness and recurrent vomiting beginning on the second day of life after an uneventful postnatal adaptation. Clinical examination of the soft abdomen did not show any abnormalities apart from the distended epigastrium. The child received intravenous fluids, and no abnormalities in laboratory blood tests were seen.

**Figure 1:**
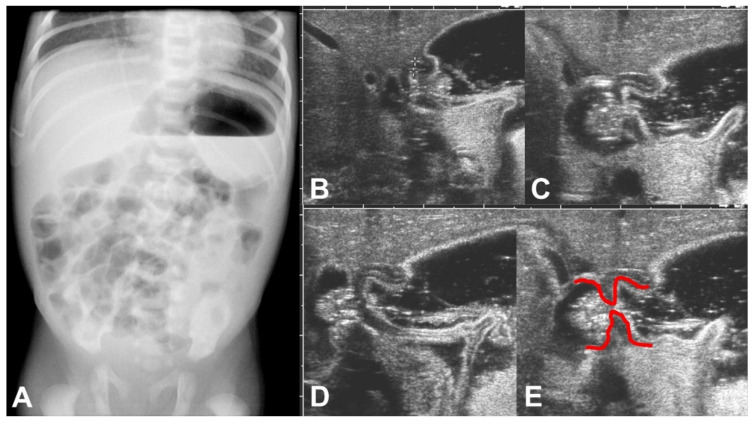
(**A**–**E**): (**A**) Erect plain abdominal X-ray on day 5 of life showed an enlarged stomach with an air-fluid level but was otherwise unsuspicious. (**B**–**E**) Sonographic examination on the same day showed a failure to pass gastric content, illustrated here in a sequence of ultrasound images during the gastric peristaltic wave. Furthermore, shouldering of the pylorus can be seen pointed out by red markings in (**E**).

On the fifth day of life, a plain abdominal X-ray showed an enlarged stomach with an air-fluid level and an unsuspicious air distribution in the small bowel loops (Figure 1A). Ultrasound examination performed on the same day confirmed the finding of the enlarged stomach and added the information of functional gastric outlet obstruction because at no time during the examination an opening of the pylorus could be seen (Figure 1B–E). The thickness of the pyloric muscle was 3 mm, and the length of the pylorus was 1.3 cm. 

**Figure 2:**
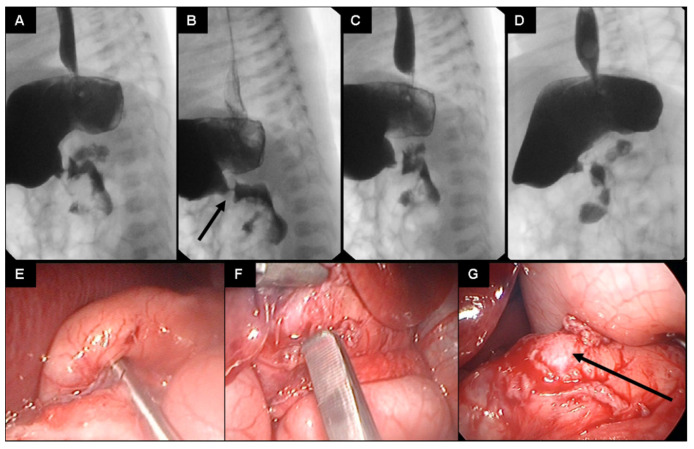
(**A**–**G**): Sequences of the upper gastrointestinal contrast study (**A**–**D**) of the presented preterm infant on day 6 of life (a water-soluble nonionic gastrointestinal radiopaque agent was used). A narrowed pyloric canal presenting as a typical “string sign” is seen (arrow in (**B**)). Only a minimal passage of the contrast agent is seen during the 20 min duration of the study. Intraoperative pictures showing congenital hypertrophic pyloric stenosis (**E**) and pyloromyotomy (**F**,**G**) (arrow pointing at pyloric mucosa).

Since the general sonographic criteria for infantile HPS were not met [1] and the child’s clinical condition did not improve, an upper gastrointestinal contrast study was scheduled for the sixth postnatal day. An extremely narrowed and non-peristaltic pylorus with only minimal contrast fluid passing through and with the typical “string sign” of HPS was found (Figure 2A–D). This led to the indication for laparoscopic exploration performed on the seventh day of life.

Abdominal access is achieved by the open technique through the upper left umbilical quadrant. A 5 mm trocar is inserted and sutured to the fascia. Pneumoperitoneum is set at 5 mmHg. A 5 mm optic is used to inspect the abdominal cavity. Trocarless insertion of the grasper in the right lateral abdomen and trocarless insertion of the knife in the left lateral abdomen. Pyloromyotomy of the thickened pyloric muscle was performed after no other pathologies were found (Figure 2E,F). The procedure is terminated when the mucosa protrudes through the incision (Figure 2G).

The postoperative course was completely uneventful. Oral feedings with breast milk were started on the first postoperative day and were well tolerated. Five days later, the patient could be sent home in good general condition weighing 2520 g. No further vomiting could be recorded, and the baby developed appropriately during the follow-up period of 13 years. The baby girl has two other female siblings from the same parents without any history of HPS. The father also never exhibited such symptoms and is internally healthy. On the other side, the mother herself presented with a large right-sided abdominal scar with a history of surgery on the fifth postnatal day due to an HPS treated by an open pyloromyotomy.

## 3. Discussion

Infantile HPS usually occurs with an incidence of 2–5 cases in 1000 births [7]. It has been reported that when the mother is affected, there is a risk of 20% for male and 7% for female offspring to also develop an HPS [7]. The mean age at diagnosis is about 38 days, and solely 0.4% of all children suffering from HPS show symptoms in the first 3 days after birth [1]. Furthermore, a decreased risk of developing HPS has been shown with increased maternal age and the number of pregnancies [1], and it is very uncommon for preterm infants to develop signs of HPS in the first week of life. Even if preterm infants may not show the typical symptoms of HPS, such as projectile vomiting and metabolic alkalosis, mild symptoms such as regurgitation are reported to occur in the first days of life in 2/3 of all cases [7]. HPS has also been reported to occur in triplets and dizygotic twins simultaneously [6,8], which was not the case in the present study.

Early-onset in a preterm infant of female gender with the mother reported to have had the same postnatal clinical course and received basically the same surgical treatment of the pyloric muscle is, to the best of our knowledge, still not reported in the literature. This case strongly supports the possibility of a hereditary cause of the congenital—early type of HPS. Ali and Haddad reported on surgical treatment of an HPS as early as 26 h after birth in a full-term baby girl and highlighted the increased incidence of congenital HPS in females [10]. The familial occurrence of HPS has been commonly reported in the literature, and genetic predisposition to the number of environmental factors identified to be associated with HPS seems to play a crucial role for usually encountered HPS between 3–12 weeks of life with male predominance [2,11]. In our case, the surgery would have been performed even earlier if there had not been confusion considering symptoms mimicking gastroesophageal reflux, which is more common in this age group. Furthermore, preterm infants have less abdominal strength to generate overt emesis, they commonly receive intravenous fluids and therefore exhibit less or no electrolyte abnormalities, and often the ultrasound measurements of pylorus do not reach the criteria for HPS reported in the literature [1,4].

We identified a small number of case reports of children being affected by HPS in the first days of life [7,10,12,13,14]. Zenn and Redo observed typical symptoms in a newborn, and an operation was performed on the fourth day of life. As a conspicuous factor, pregnancy has been complicated by polyhydramnion in their case [12]. Houben and Kiely reported a preterm baby developing symptoms of HPS on the second day of life. Their case was also associated with polyhydramnion, and they speculated that there might be coherence between congenital HPS and polyhydramnion [13]. In our case, no polyhydramnion was noted during the pregnancy.

**Figure 3:**
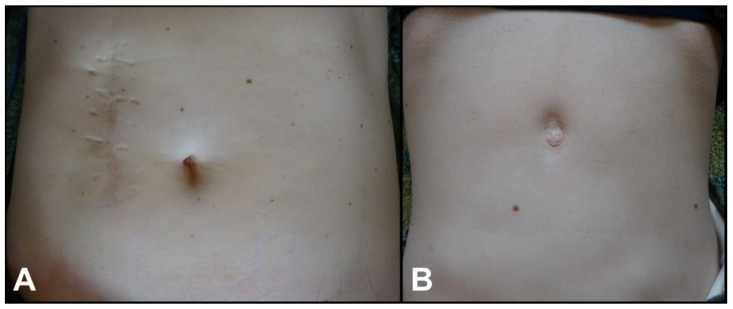
(**A**,**B**): (**A**) Cosmetic appearance of the abdomen of the mother at the age of 45 years after laparotomy for congenital hypertrophic pyloric stenosis (CHPS) on day 5 of life. (**B**) Cosmetic appearance of the abdomen of her daughter at the age of 13 years after laparoscopic pyloromyotomy for CHPS on day 7 of life. Both had an uneventful postoperative period and are healthy at the time of this report.

Because standard ultrasound criteria for the measurement of pyloric muscle size in children with HPS may not be met in preterm infants, and also these criteria may not be valid for children with congenital HPS, an upper gastrointestinal contrast study has been used by others [10,13] and in the present study to confirm the diagnosis of HPS. Although the etiopathogenesis of HPS may be different, the treatment of HPS being congenital or infantile is the same. The relief of symptoms is established after a classic pyloromyotomy is performed through nowadays more preferable laparoscopic technique or by using any of the reported open surgical techniques [15,16]. We treated our case by laparoscopy and had an uneventful outcome. The mother of the child was treated by open technique in the pre-laparoscopic era and also had an uneventful outcome. However, the cosmetic results are significantly different (Figure 3).

Although several susceptible gene loci have been reported, no specific gene that would be responsible for the development of HPS has yet been identified [9,17]. In addition, a number of hypotheses concerning the etiology of HPS have been established, such as a failure of production of neuronal nitric oxide synthase (nNos) based on the critical role of this enzyme in the production of nitric oxide, which is involved in the processes of relaxation and contraction of the pyloric muscle [18,19]; the loss of peptide immunoreactivity among the nerve fibers of the circular pyloric muscle appearing in less than 5% of normal values in HPS patients [20]; the reduced density of nerve terminals and neurofilaments in the pyloric muscle layer resulting in poor neuronal innervation [21]; the nearly complete absence of interstitial cells of Cajal, which are replaced by resembling cells representing a failure in the maturational process of the pyloric muscle [9,22]; and last but not least hyperacidity due to an enlarged mass of parietal cells [23]. Babies secrete the hormone gastrin independently at birth, and they reach even higher levels of the hormone than adults when fasting. Repeated hyper acidic stimulation evokes repeated pyloric sphincter contraction with work hypertrophy and vomiting [23].

In summary, HPS is an entity that must be accounted for in both term and preterm newborns with recurrent non-bilious postnatal vomiting and a failure to thrive. These cases are rare and should be gathered for future research as they could have different etiopathogenesis when compared to common infantile HPS occurring between the third and twelfth postnatal week. To the best of our knowledge, this is the first study reporting the occurrence of congenital HPS in a mother and her child in their first week of life.

## Data Availability

The raw data supporting the conclusions of this article will be made available by the authors, without undue reservation.
